# Compound Heterozygous Mutations in *TMC1* and *MYO15A* Are Associated with Autosomal Recessive Nonsyndromic Hearing Loss in Two Chinese Han Families

**DOI:** 10.1155/2020/8872185

**Published:** 2020-08-01

**Authors:** Pengcheng Xu, Jun Xu, Hu Peng, Tao Yang

**Affiliations:** ^1^Department of Otorhinolaryngology-Head and Neck Surgery, Shanghai Ninth People's Hospital, Shanghai Jiao Tong University School of Medicine, Shanghai, China; ^2^Ear Institute, Shanghai Jiao Tong University School of Medicine, Shanghai, China; ^3^Shanghai Key Laboratory of Translational Medicine on Ear and Nose Diseases, Shanghai, China; ^4^Department of Otolaryngology-Head and Neck Surgery, Changzheng Hospital, Second Military Medical University, Shanghai, China

## Abstract

Genetic hearing loss is a common sensory disorder, and its cause is highly heterogeneous. In this study, by targeted next-generation sequencing of 414 known deafness genes, we identified compound heterozygous mutations p.R34X/p.M413T in *TMC1* and p.S3417del/p.R1407T in *MYO15A* in two recessive Chinese Han deaf families. Intrafamilial cosegregation of the mutations with the hearing phenotype was confirmed in both families by the Sanger sequencing. Auditory features of the affected individuals are consistent with that previously reported for recessive mutations in *TMC1* and *MYO15A*. The two novel mutations identified in this study, p.M413T in *TMC1* and p.R1407T in *MYO15A*, are classified as likely pathogenic according to the guidelines of ACMG. Our study expanded the mutation spectrums of *TMC1* and *MYO15A* and illustrated that genotype-phenotype correlation in combination with next-generation sequencing may improve the accuracy for genetic diagnosis of deafness.

## 1. Introduction

Congenital hearing impairment is a common birth defect worldwide, occurring in approximately 1-2 per 1000 infants. With increasing age, the prevalence continues to rise to 2.7 per 1000 before age five and 3.5 per 1000 through adolescence [1]. To date, more than 100 genes have been reported to be associated with nonsyndromic hearing loss (NSHL), including 76 autosomal recessive nonsyndromic hearing loss (ARNSHL) genes, 48 autosomal dominant nonsyndromic hearing loss (ADNSHL) genes, and 5 X-linked nonsyndromic hearing loss genes (Hereditary Hearing Loss Homepage; https://hereditaryhearingloss.org/, updated in January 2020). Hair cells (HCs) in the cochlea mainly function in converting the sound mechanical waves into the electric neural signals [2–4] which make it extremely critical for the hearing ability. Many previous studies have shown that HCs can be damaged due to genetic factors, ototoxic drugs, noise, inflammation, or aging, among which genetic account for 50% of the HC malfunction [5–11].

The *TMC1* gene is located on chromosome 9q21 and contains 24 exons that encodes a 760 amino acid membrane protein TMC1 with six transmembrane domains [12, 13]. TMC1 is a pore-forming subunit of the mechanotransduction complex that is predicted to have transmembrane domains with intracellular N and C termini and one conserved TMC domain [14]. TMC1 is expressed in the mouse inner ear and has been suggested to involve in the functional maturation and survival of cochlear HCs [12]. It has been reported that mutations in *TMC1* may cause both prelingual profound autosomal recessive deafness DFNB7/11 and postlingual progressive autosomal dominant deafness DFNA36 [13]. To date, more than 60 mutations in *TMC1* are reported worldwide [15], with the recessive mutations predominantly associated with prelingual severe-to-profound hearing loss [15, 16].

The HC stereocilia is critical to maintain the function of HC [17, 18]. The *MYO15A* gene is located at chromosome 17p11.2 and contains 66 coding exons, which encodes an unconventional myosin protein Myosin XVA [19]. Myosin XVA is a large actin-based motor protein. In cochlear hair cells, it is critical for elongation and differentiation of the stereocilia [20]. Myosin XVA displays an important role in the mechanotransduction of cochlear hair cells. Myosin XVA through its carboxy-terminal PDZ-ligand interacts with the third PDZ domain of whirlin, and then delivers whirlin to the tips of stereocilia [21]. *MYO15A* mutations are responsible for congenital deafness DFNB3 in human and cochleovestibular dysfunction in shaker 2 mice which shows abnormally short stereocilia bundles and diminished staircase [20, 21]. It is one of the most common causes of ADNSHL in Mideast countries due to prevalent consanguineous marriage [22, 23], with majority associated with prelingual severe-to-profound hearing loss and mutations in exon 2 leading to a milder auditory phenotype [23].

In this study, we presented the clinical characterization and genetic analysis of two Chinese Han families affected by ARNSHL. Using targeted next-generation sequencing of 414 known deafness genes, we identified compound heterozygous mutations in *TMC1* and *MYO15A* as the genetic causes of the hearing loss in those families.

## 2. Materials and Methods

### 2.1. Subjects and Clinical Evaluations

**Figure 1:**
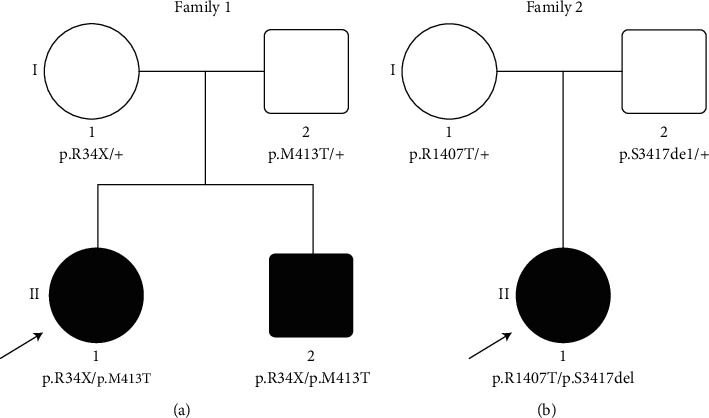
Pedigrees and genotypes of Family 1 (a) and Family 2 (b).

This study included two nuclear Chinese Han recessive deaf families: Family 1 (Figure 1(a)) and Family 2 (Figure 1(b)). All affected family members underwent clinical evaluation in the Department of Otolaryngology-Head and Neck Surgery, Shanghai Ninth People's Hospital, Shanghai Jiao Tong University School of Medicine, Shanghai, China. The evaluation included a complete medical history interview and a comprehensive physical examination including otoscopic examination to exclude hearing loss due to infections, trauma, or other environmental factories. Middle ear function was evaluated through tympanometry, and the function of the outer hair cells of the cochlea was evaluated by distortion production otoacoustic emissions (DPOAE). Pure-tone audiometry (PTA) was calculated as the average of the hearing threshold of patients at 500, 1,000, 2,000, and 4,000 Hz. The degree of hearing loss was defined as mild (26–40 dB HL), moderate (41–55 dB HL), moderately severe (56–70 dB HL), severe (71–90 dB HL), and profound (>90 dB HL). Hearing thresholds reported in this study were averaged air-conducted pure-tone thresholds of each side. Tandem gait and the Romberg testing were performed for vestibular function examination. Computerized tomography (CT) scan of the temporal bone was carried out to assess the development of the anatomical structures of the middle and inner ear for the available subjects. This study was approved by the ethnic committee of Shanghai Ninth People's Hospital. Written informed consents were obtained from each participant or from parents of the young subject.

### 2.2. Mutation Identification

The genomic DNA of peripheral blood was extracted from all subjects using a Blood DNA kit according to the standard protocol (QIAamp DNA Blood Mini Kit, QIAGEN, Shanghai). Targeted next-generation sequencing was performed as previously reported [24, 25]. The exons, splicing sites, and flanking intronic region of 414 known deafness-related genes (Table S1) were captured by a customized capture assay (MyGenostics, Beijing, China). Candidate pathogenic mutations were defined as nonsynonymous (including nonsense, missense, splice-site, and indels) variants that had allele frequencies under 0.01 in the 1000 Genomes database, the dbSNP database, the Exome Aggregation Consortium database (ExAC), and data from 200 Chinese Han normal-hearing control individuals. The potential pathogenic effects of candidate mutations were evaluated by in silico tools Mutation Taster, SIFT, and PolyPhen2. Cosegregation of the disease phenotype and the causative mutation was confirmed in all family members by PCR amplification and the Sanger sequencing. Pathogenicity of the mutations were classified following the guidelines of ACMG 2015 [26].

## 3. Results

### 3.1. Clinical Characterization

#### 3.1.1. Family 1

**Figure 2:**
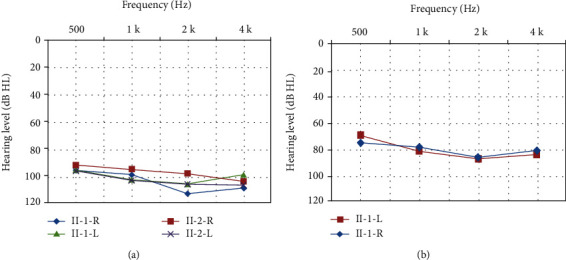
Audiograms of the affected members of Family 1 (a) and Family 2 (b).

Family 1 has two affected siblings born to two normal-hearing parents (Figure 1(a)). The proband II-1 was a 26-year-old female with congenital sensorineural hearing loss. Tympanogram indicated normal function of the middle ear. Bilateral DPOAE were absent. Both II-1 and her younger brother II-2 suffered from bilateral, profound hearing impairment with PTA thresholds above 90 dB HL (Figure 2(a)). Tandem gait and the Romberg testing displayed no symptoms of vestibular dysfunction. Temporal bone CT scans showed no obvious abnormalities. No apparent additional syndromic features were found.

#### 3.1.2. Family 2

The proband II-1 of Family 2 (Figure 1(b)) was a 10-year-old girl, who suffered from prelingual bilateral hearing impairment. Auditory examination and PTA showed the sensorineural hearing loss is severe (Figure 2(b)). Tympanogram displayed a type A curve, indicating normal function of the middle ear. Bilateral DPOAE were absent, and no vestibular dysfunction was recorded for the proband. No other abnormality was discovered by the medical history and physical examination.

### 3.2. Mutation Analysis

**Figure 3:**
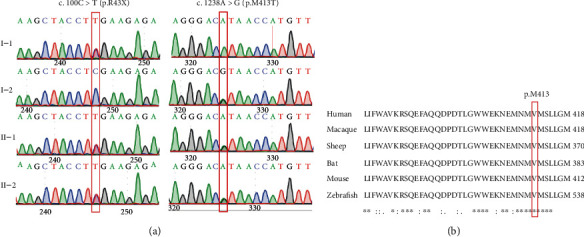
(a) The Sanger sequencing results of the p.R34X and p.M413T mutations in *TMC1* in Family 1. (b) Multispecies sequence alignment of the M413 residue.

Targeted next-generation sequencing of 414 known deafness genes was performed for the probands Family1-II-1 and Family2-II-1. A total of 9 and 13 candidate variants were identified, respectively (Table S2). In Family 1, compound heterozygous mutations c.100C>T (p.R34X) and c.1238T>C (p.M413T) in *TMC1* (NM_138691) were identified as the only candidate mutations consistent with the recessive inheritance. The Sanger sequencing revealed that the mutations cosegregated with the hearing phenotype in Family 1, as the unaffected parents, were heterozygous carriers of single mutations p.R34X (mother I-1) and p.M413T (father I-2), while the affected siblings both had compound heterozygous mutations (Figure 3(a)). These two mutations were not detected in 200 Chinese Han normal-hearing controls and are not present in 1000 Genomes and ExAC databases. The p.R34X mutation with minor allele frequency (MAF) of 0.0002 in ExAC has been previously detected in many patients from Pakistan, Iran, Turkey, and Tunisia but is relatively rare in China [16, 27–29]. On the other hand, while the p.M413T mutation is novel. Based on the ACMG guidelines, the p.R34X and p.M413T mutations in *TMC1* were classified as pathogenic (PVS1+PS1+PM2+BS2) and likely pathogenic (PM2+PM3+PP3), respectively.

**Figure 4:**
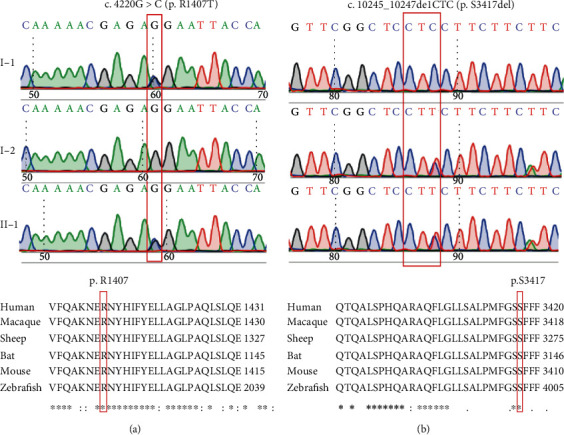
(a) The Sanger sequencing results of the p.S3417del and p.R1407T mutations in *MYO15A* in Family 2. (b) Multispecies sequence alignment of the S3417 and R1407 residues.

In Family 2, compound heterozygous variants c.10245_10247delCTC (p.S3417del) and c.4220G>C (p.R1407T) in *MYO15A* (NM_016239) were considered the only candidate pathogenic variants consistent with the recessive inheritance. The Sanger sequencing confirmed that the mutations cosegregated with the hearing phenotype in Family 2, as the unaffected parents were heterozygous carriers of single mutations p.R1407T (mother I-1) and p.S3417del (father I-2) (Figure 4(a)). These two mutations were not detected in 200 Chinese Han normal-hearing controls and are not present in 1000 Genomes and ExAC databases. The p.S3417del mutation with MAF of 0.000016 in ExAC has been previously reported to cause autosomal recessive hearing loss in Japanese and Korean patients, but not in the Chinese population [30, 31]. The p.R1407T mutation is novel. Based on the ACMG guidelines, the p.S3417del and p.R1407T mutations in *MYO15A* were classified as pathogenic (PS1+PM2+PM3+PM4) and likely pathogenic (PM2+PM3+PP3), respectively.

## 4. Discussion

Recessive hearing loss accounts for the majority (80%) of genetic hearing loss [32]. Among many genes responsible for ARSNHL, mutations in *GJB2* are the most frequent causes [33, 34], followed by that in *SLC26A4*, *TMC1*, and *MYO15A* especially in Middle East countries where consanguineous marriage is common [15, 22, 35]. On the contrary, in China where consanguineous marriage is far less frequent, recessive mutations in *TMC1* and *MYO15A* were not as extensively reported in the literature.

**Figure 5:**
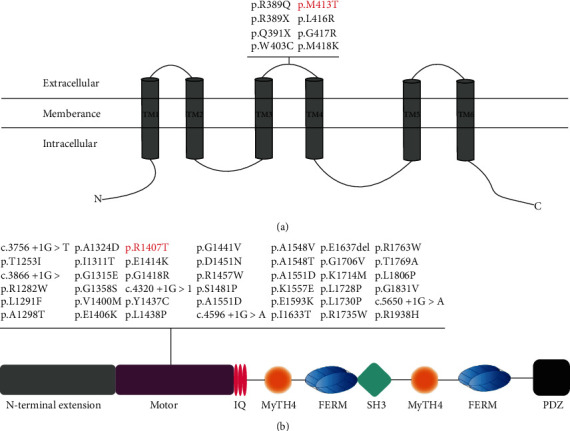
(a) Transmembrane domain structure of TMC1 and mutations in the second extracellular loop of *TMC1*. The novel p.M413T mutation identified in this study is in red. (b) Protein structure of Myosin XVA and missense mutations in the motor domain. The novel p.R1407T mutation identified in this study is in red.

In Family 1, we identified compound heterozygous mutations p.R34X and p.M413T in *TMC1*. The p.R34X mutation is the most common *TMC1* mutation in Pakistan [36]. Using polymorphic markers, Ben Said et al. showed that this nonsense mutation is an old founder mutation emerged between the years 1075 and 1900 along with the third Hadhramaut population movements [27]. This nonsense mutation is predicted to produce a prematurely truncated protein and is associated with congenital, severe-to-profound deafness [13, 27]. The p.M413T mutation identified in this study has not been previously reported. It is predicted as deleterious by computational tools PolyPhen2 and SIFT. The p.M413T mutation is located in the second extracellular loop between the third and fourth transmembrane domains of TMC1, and the methionine 413 residue is well conserved among different species (Figure 3(b)). At least seven mutations in the second extracellular loop, including five missense mutations, have already been associated with ARNSHL and ADNSHL (Figure 5(a)), suggesting an important role of this particular region in the inner ear function of TMC1.

In Family 2, we identified compound heterozygous mutations p.S3417del and p.R1407T in *MYO15A*. The p.S3417del mutation deletes a serine3417 residue at the second FERM domain of Myosin XVA. The FERM domain is a protein-binding domain important in cargo transport and cytoplasmic protein connection to the membrane [37, 38]. This mutation has been previously reported in Japanese and Korean deaf patients but not in the Chinese population [30, 31]. The novel p.R1407T mutation identified in this study is predicted as deleterious by computational tools PolyPhen2 and SIFT. This mutation is located in the motor domain of Myosin XVA, which is next to the long N-terminal extension and is highly conserved among different species (Figure 4(b)). To date, more than 40 missense mutations in the motor domain of *MYO15A* have been associated with ARSNHL (Figure 5(b)). The motor domain is essential for ATP activity and possesses two binding sites for actin and ATP, which can produce force to move the actin filaments. In the mouse model, *MYO15A* mutation in the motor domain results shorter stereocilia with an abnormal staircase structure [39].

Considering the high degree of genetic heterogeneity, the next-generation sequencing (NGS) technology has been proven an effective method for genetic testing of hearing loss in recent years. However, previous studies have showed that NGS in deaf patients, especially the sporadic cases, may detect a significant amount of rare, nonsynonymous variants with unknown functional significance and sometimes even results in false-positive diagnosis [25]. In this study, we obtained a detailed hearing phenotype for all patients, which is consistent with those from previous reports for ARSNHL patients with recessive *TMC1* and *MYO15A* mutations. Our data suggested that the genotype-phenotype correlation may facilitate more accurate genetic diagnosis of deafness in such cases.

## 5. Conclusions

Compound heterozygous mutations p.R34X/p.M413T in *TMC1* and p.S3417del/p.R1407T in *MYO15A* were identified as the pathogenic causes of ARSNHL in two Chinese Han families. Our results expanded the mutation spectrum of those two genes and showed that NGS in combination with genotype-phenotype correlation may provide a more accurate diagnosis for genetic deafness.

## Data Availability

The data used to support the findings of this study are available from the corresponding authors upon request.

## Supplementary Materials

Supplementary Materials Table S1: the 414 deafness-related genes sequenced by targeted NGS. Table S2: candidate mutations identified in probands F1-II-1 and F2-II-1 by targeted NGS.
